# Description of an App-Based Telerehabilitation Platform After Stroke in India (ATTEND-2) Using the Template for Intervention Description and Replication (TIDieR) Checklist

**DOI:** 10.7759/cureus.110294

**Published:** 2026-06-05

**Authors:** Rinita Mascarenhas, Anuj G Nair, Mohammed Alfaz, Angel C Selvaraj, John M Solomon, Jeyaraj D Pandian, Dorcas Gandhi

**Affiliations:** 1 Department of Neurology, Christian Medical College & Hospital, Ludhiana, IND; 2 Department of Technology, Buildreams Technologies, Udupi, IND; 3 Department of Physiotherapy, Manipal College of Health Professions, Manipal Academy of Higher Education, Manipal, IND; 4 Department of Physiotherapy, Christian Medical College & Hospital, Ludhiana, IND

**Keywords:** contextualization, india, stroke rehabilitation, telerehabilitation, upper limb

## Abstract

Stroke rehabilitation in India is challenged by shortages of rehabilitation professionals and limited access to structured post-discharge care, particularly in rural settings. Although family-led rehabilitation approaches have demonstrated feasibility in increasing therapy engagement, there remains a need for scalable systems that support continuity, monitoring, and accessibility of rehabilitation services. ATTEND-2 was developed as a culturally contextualized, app-based telerehabilitation platform to support post-stroke upper limb rehabilitation in resource-constrained settings. This study aimed to describe the ATTEND-2 intervention using the Template for Intervention Description and Replication (TIDieR) checklist. The intervention was systematically developed through stakeholder engagement involving stroke survivors, caregivers, physiotherapists, neurologists, and software developers. The updated Consolidated Framework for Implementation Research (CFIR) domains were applied to characterize implementation determinants influencing adoption and scalability within the Indian health system. The resulting intervention supports remote supervision, structured exercise prescription, and adherence monitoring within Indian home environments. CFIR-guided analysis identified key facilitators for scalability, including adaptability across urban and rural settings, compatibility with task-sharing approaches, and alignment with India’s evolving digital health ecosystem. ATTEND-2 represents an evidence-informed telerehabilitation intervention designed to strengthen access to post-stroke upper limb rehabilitation. Structured reporting using TIDieR-telehealth and CFIR enhances transparency, reproducibility, and future feasibility and effectiveness evaluation.

## Introduction

Stroke rehabilitation remains a major unmet need in low- and middle-income countries (LMICs), where most survivors live with long-term disability and limited access to post-acute care. In India, which accounts for 13%-15% of the global stroke burden with 1.8 million annual cases, 9.34 million years lived with disability, and a death every 4 minutes, organized services reach fewer than 3% of the 1.25 million new survivors yearly amid stark therapist shortages (1 per 176,000 population). Pronounced rural-urban disparities exacerbate this crisis, as infrastructure deficits leave 82% of survivors and caregivers without specialist therapy after discharge, underscoring the urgent need for scalable, family-led interventions [1-4].

The ATTEND trial (2017) represented a landmark effort to address these gaps through a family-led rehabilitation model, demonstrating that caregiver involvement could significantly increase therapy time [5]. However, the trial did not show significant improvements in death, dependency, or functional outcomes, underscoring the limitations of unsupervised, informal caregiving without structured guidance, monitoring, and behavioral reinforcement. Alternatively, telerehabilitation has emerged as a promising strategy for improving access, adherence, and continuity of “monitored” stroke rehabilitation [6,7]. Global evidence supports the effectiveness of telerehabilitation as an adjunct for improving motor function and participation, yet India-specific implementations remain scarce and insufficiently contextualized [8,9]. Existing Indian applications, such as "Care for Stroke," primarily emphasize education and awareness; preliminary results reported no significant improvement in mobility [10].

Critical contextual challenges, including limited digital literacy, variable smartphone access in rural populations, multilingual needs, gender inequities in caregiving, and high stroke recurrence rates, further constrain the effectiveness of generic digital solutions [11,12]. ATTEND-2 addresses these challenges through a mixed-methods participatory approach to develop a low-resource, app-based telerehabilitation platform co-designed with stroke survivors, caregivers, and therapists. Unlike the ATTEND family-led rehabilitation model, ATTEND-2 incorporates structured therapist support, remote monitoring, and behavioral reinforcement strategies to improve adherence and continuity of rehabilitation within low-resource Indian settings [5].

Therefore, this paper describes the development and intervention design of the ATTEND-2 app-based telerehabilitation program for post-stroke upper-limb rehabilitation in India, using the Template for Intervention Description and Replication (TIDieR) checklist.

## Technical report

Intervention description framework

This Methods section outlines the reporting framework used to describe the ATTEND-2 intervention based on the TIDieR-telehealth extension checklist [13] and the Neuro-Rehabilitation OnLine [14] program. These frameworks were used to ensure transparent and reproducible reporting of a complex, home-based telerehabilitation intervention. Intervention content and checklist components were developed through a collaborative process informed by existing evidence and stakeholder input. Stakeholders included stroke survivors, caregivers, physiotherapists, neurologists, and software developers, reflecting the multidisciplinary nature of stroke rehabilitation and digital health delivery in the Indian context. Details of the collaborative, stakeholder-informed development process underpinning the intervention content and checklist components are reported in a separate publication. Given the close interaction between complex interventions and the systems within which they are delivered, the implementation context for ATTEND-2 was examined using domains of the updated Consolidated Framework for Implementation Research (CFIR) [15] (provided in Appendix A). This framework was applied to systematically describe inner and outer setting factors influencing the potential adoption, implementation, and scale-up of home-based telerehabilitation in India. The app and exercises are currently undergoing the copyright process.

Digital platform and materials development

The ATTEND-2 app is developed using Flutter, a cross-platform framework enabling deployment on both Android and iOS devices. The backend uses Firebase to support secure authentication, real-time data synchronization, and scalable cloud storage, facilitating efficient handling of patient data and enabling real-time updates, reminders, and notifications. A non-relational database is used for storing and retrieving user and exercise data. Server hosting and maintenance requirements have been estimated based on anticipated initial usage (approximately 50 patients and 300 total users). The app integrates third-party services for video consultations and push notifications, with resource requirements estimated based on anticipated usage patterns.

Platform functionality

The platform comprises three user modules -- patient, therapist/doctor, and administrator -- each with specific access controls and functions (Table 1).

**Table 1:** Overview of the ATTEND-2 App Modules: Admin, Therapist, and Patient FMA-UE, Fugl-Meyer Assessment for upper extremity.

Patient module	Therapist module	Admin module
Personalized weekly exercise plan with sets, repetitions, and goals. Exercise and activity-based videos recorded with stroke survivors, tailored to Indian home environments. Instructions available in English, Punjabi, and Kannada. Safety icons and prompts before each exercise. Progress dashboard with activity logs, adherence streaks (plant-growth metaphor), and a calendar view. Automated reminders	Ability to assign or modify personalized weekly therapy plans. Access to the complete exercise library, categorized by upper limb impairment severity (based on FMA-UE). Integrated video consultations enabling synchronous remote assessment, complemented by asynchronous exercise delivery and progress monitoring. Automated progress charts displaying adherence and performance trends over time. E-report generation summarizing patient assessments, prescribed exercises, adherence, and performance outcomes. Access to patient health profiles, assessment records, individualized therapy plans, and progress dashboards	User management (including enrollment and de-enrollment of patients and therapists). Editing and updating exercise videos, educational content, and app materials. Monitoring real-time usage and system analytics. Oversight of activity logs and system integrity. Ability to generate utilization and performance reports for monitoring and evaluation

Intervention content

The ATTEND-2 intervention involves a structured, contextualized home-based exercise program delivered remotely via a smartphone application. A comprehensive set of upper limb exercises commonly prescribed after stroke was identified through a review of the published literature and existing exercise repositories previously used in Indian stroke rehabilitation research. These included exercise materials from the ATTEND manual, stroke rehabilitation exercise sets described in prior Indian studies, physical activity lists developed through a doctoral research program in India, and home-based rehabilitation exercise protocols from related telerehabilitation studies, refined and categorized into mild, moderate, and severe impairment levels based on Fugl-Meyer Assessment for upper extremity (FMA-UE) criteria [16-18]. Contextualization of the exercise content was guided by the following considerations: (i) Adaptation to home environments: Exercises were selected and modified to be performed safely in small, crowded, or shared household spaces common in urban and rural Indian settings. (ii) Minimal equipment design: All tasks required no specialized therapy equipment. (iii) Use of familiar household objects: Everyday items, such as towels, water bottles, utensils, chairs, and walls, were incorporated to replace clinic-based tools and promote easy practice at home. (iv) User-informed co-development: Stroke survivors, caregivers, and physiotherapists contributed feedback through iterative testing cycles, ensuring that the exercises were culturally acceptable, meaningful, and aligned with real-world home constraints (Appendices B-D provide detailed descriptions of all exercises).

Delivery team

The ATTEND-2 intervention can be delivered by a multidisciplinary team of healthcare providers, supporting patient rehabilitation through both remote and in-person guidance. Each professional group contributes specialized expertise and receives targeted training to effectively deliver the telehealth components, such as: (i) Rehabilitation therapists (physiotherapists/occupational therapists): Licensed therapists with experience in stroke rehabilitation, neurorehabilitation principles, and functional training. Providers typically hold a bachelor’s or master’s degree in physiotherapy or occupational therapy. (ii) Medical doctors (physical medicine and rehabilitation/neurology/general medicine): Physicians involved in stroke care with competencies in diagnosis, medical stabilization, and rehabilitation planning. They can refer patients to the app, enabling therapists to provide exercise oversight and monitor medical safety. (iii) Nursing staff/community health workers (CHWs): Nursing assistants or CHWs engaged in patient monitoring, caregiver education, and reinforcement of home-based rehabilitation routines.

Training for telehealth delivery

Providers across all categories will receive structured preparation to safely and effectively deliver the ATTEND-2 telehealth intervention. Therapists will be trained in remote movement assessment, telecoaching methods, safety monitoring, and identifying home environment risks, skills that differ from hands-on, in-person facilitation. Administrative staff will be trained to navigate the admin and patient modules, conduct adherence checks, manage remote follow-ups, and recognize red flags, compensating for the absence of face-to-face interaction with enhanced communication and escalation protocols. The ATTEND-2 app will be installed on the patient’s or caregiver’s smartphone. Following registration, a demonstration is provided to ensure correct and safe use of the application for rehabilitation purposes.

Mode of delivery and setting

The intervention will be delivered entirely through telehealth using the ATTEND-2 platform, combining both synchronous and asynchronous formats. Synchronous sessions included one-on-one video consultations between the therapist and patient for assessment, exercise progression, safety checks, and problem-solving. Asynchronous components included pre-recorded exercise videos, daily reminders, progress-tracking dashboards, and messaging features for therapist feedback. Therapists will conduct asynchronous reviews of patient logs, exercise adherence, and flagged alerts, with optional brief video consultations when clinical clarification is required. All rehabilitation will be provided individually.

The intervention will be delivered remotely, with both patients and clinicians operating from locations that support effective telehealth use. Patients will perform exercises as prescribed by their therapists at home. Caregivers may assist in each session. Clinicians (therapists and doctors) will deliver care from their workstations within the partnering hospitals or rehabilitation centers. These workstations will be equipped with secure high-speed internet, privacy-compliant surroundings, and devices optimized for video assessment.

All asynchronous components (e.g., daily exercise videos, adherence checks, and progress logs) will occur within the patient's home environment, while synchronous components (live video sessions and teleconsultations) will be conducted from the clinician’s telehealth workstation. 

Intervention and dose

At the time of recruitment, each participant will be asked to indicate their preferred time window for scheduling therapy interactions, allowing flexibility and alignment with individual routines. The application sends notifications to the patient or caregiver within this preferred time window to support adherence.

The intervention is delivered over a 12-week period, combining structured, asynchronous, home-based exercise sessions with scheduled, synchronous therapist consultations. Participants will receive up to 45 minutes of exercise per session, 5 days per week, delivered through asynchronous video-guided modules within the application. Each exercise video lasts a maximum of two minutes and can be replayed as needed to achieve the prescribed repetitions, based on the participant’s impairment severity, tolerance, and fatigue levels.

In addition, participants will receive three synchronous teleconsultation sessions per week with a therapist. These sessions are designed to review performance, address challenges, modify exercise prescriptions, and provide individualized feedback and motivational support.

The overall exercise dose and progression are supported through app-generated reminders, automated activity tracking, and therapist review of weekly performance logs, ensuring that the prescribed intensity and frequency of rehabilitation are maintained throughout the intervention period.

Tailoring and modifications

The ATTEND-2 intervention is designed to allow tailoring based on the individual’s level of impairment and progression over time. Exercises are initially selected according to FMA-UE-based severity categories (mild, moderate, and severe). The description of the exercises is provided in Appendices E, B, and C. Ongoing adjustments to exercise selection, intensity, and frequency are guided by therapist review of app-generated progress logs and patient-reported challenges, as part of routine intervention delivery. 

This article describes the ATTEND-2 innovation, contextualized for the Indian context. Further modifications will be included after a six-week intervention period and the inclusion of adult stroke survivors at varying severity of stroke.

Fidelity

To ensure fidelity of intervention delivery, several monitoring strategies are incorporated within the platform. The application automatically records exercise completion, frequency of engagement, and duration of activity sessions. Therapists review these logs weekly through the clinician dashboard to verify adherence. Synchronous teleconsultations provide an opportunity for therapists to directly observe patient performance and reinforce support strategies. Fidelity to the planned protocol is further supported through standardized exercise libraries categorized by impairment level and structured therapist workflows embedded within the platform.

In addition, therapists participating in ATTEND-2 undergo orientation sessions covering teleassessment techniques, remote coaching strategies, and safe progression of home-based exercises. These sessions ensure consistency in intervention delivery across providers and reduce variability in remote rehabilitation practices. At the time of recruitment, stroke survivors and/or their caregivers are trained to use the application, including navigating exercise modules, responding to reminders, and logging activity within the platform.

Fidelity is further enhanced through a gamification component integrated into the application, where users are rewarded with a visual “tree growth” feature that progresses with consistent exercise completion. This serves as a motivational tool to encourage adherence and sustained engagement with the rehabilitation program.

Implementation context

The ATTEND-2 intervention will be delivered within the Indian setting, encompassing tertiary hospitals and community-based rehabilitation services. Participants will be adult stroke survivors across varying severities, with caregivers actively engaged to support home-based rehabilitation. Urban-rural differences, household infrastructure constraints, and varying levels of digital literacy will be considered during intervention delivery. 

**Figure 1:**
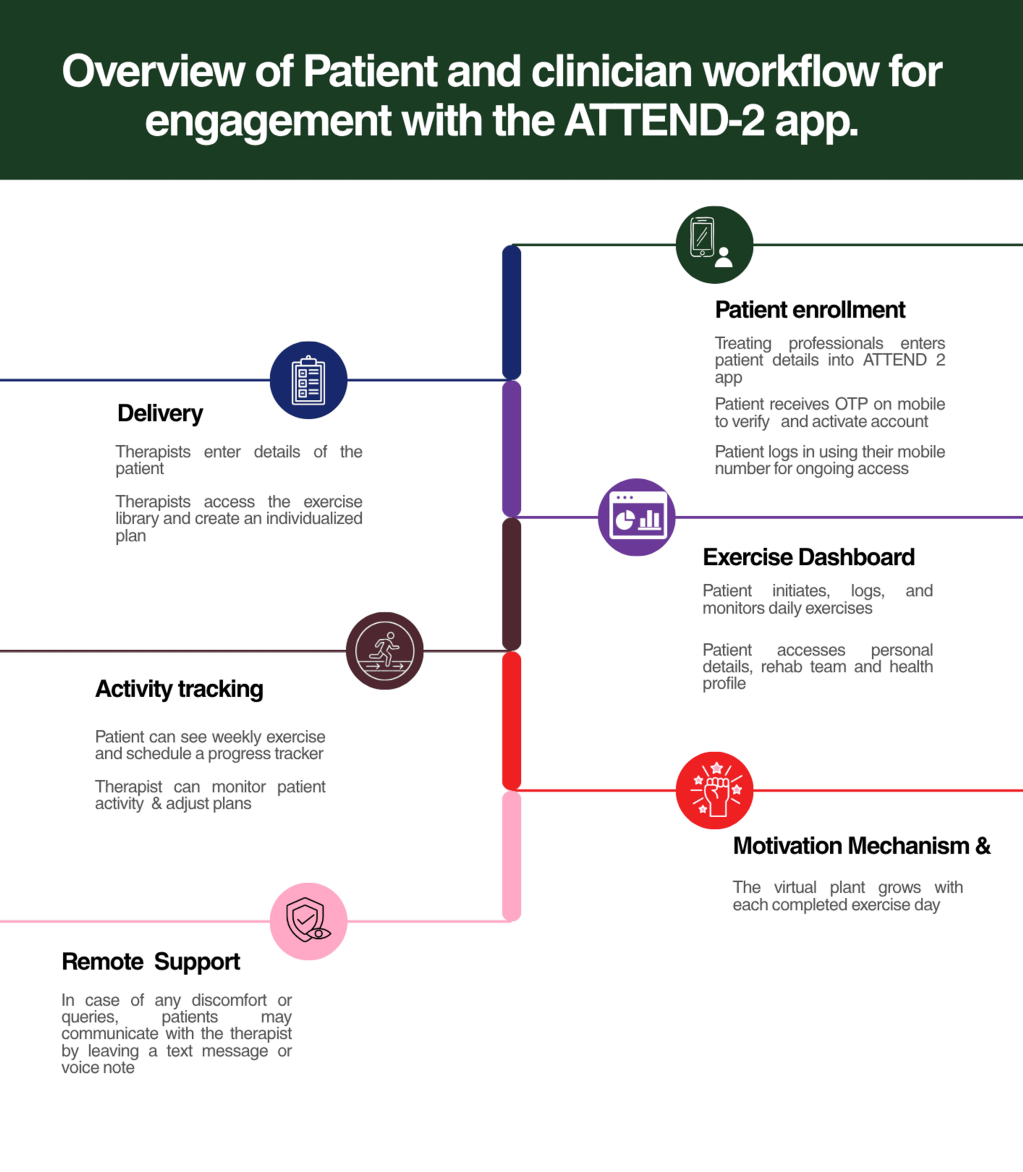
Patient-Therapist Workflow for Synchronous and Asynchronous Engagement in the ATTEND-2 App The figure has been created using Canva.

The intervention is implemented through hospitals and rehabilitation centers, with existing healthcare staff (therapists, nurses, and doctors) trained to deliver the intervention remotely. Resource availability, staffing levels, and access to smartphones and internet connectivity influence implementation fidelity (Figure 1).

## Discussion

Stroke is a relatively common and potentially life-threatening neurological condition, most often caused by the formation of a thrombus within cerebral blood vessels [19]. The ATTEND-2 app-based telerehabilitation intervention comprises user-friendly digital modules incorporating adherence strategies such as automated notifications, personalized goal-setting, and real-object therapy. It is described in this paper using the TIDieR-telehealth checklist with explicit consideration of implementation within the Indian context. By prioritizing transparent reporting and contextual adaptation, ATTEND-2 facilitates scalable service delivery. In a country where organized post-stroke rehabilitation reaches only a small proportion of survivors and therapist shortages remain substantial, digitally enabled task-sharing models are essential rather than optional.

The original ATTEND trial demonstrated that family-led rehabilitation was safe and acceptable but did not significantly improve survival or functional outcomes, underscoring the limitations of largely unsupervised care [5]. ATTEND-2 addresses this gap by integrating structured therapist oversight, behavioral reinforcement, and real-time monitoring within a telehealth framework. The platform introduces structured exercise prescription based on impairment severity, automated adherence monitoring, synchronous teleconsultations, progress dashboards, and behavioral nudges designed to promote sustained engagement. In addition, ATTEND-2 incorporates culturally contextualized exercise demonstrations and multilingual interfaces tailored to Indian home environments. By integrating digital supervision within a task-sharing model, ATTEND-2 moves beyond unsupervised caregiver support and provides a hybrid model of monitored home-based rehabilitation. This approach contributes new knowledge on how digital health platforms can strengthen family-led rehabilitation models in low-resource settings while maintaining therapist oversight.

From an implementation perspective, ATTEND-2 aligns with existing Indian health system realities. The use of smartphones, minimal equipment, multilingual content, and culturally contextualized exercise videos reduces the most common barriers. The three-tiered architecture (patient, therapist, and administrator) is similar to institutional hierarchies, facilitating adoption within tertiary hospitals and community rehabilitation services. Importantly, ATTEND-2 moves beyond the passive provision of exercise videos by embedding remote supervision, defined communication pathways, monitoring systems, and workflow adaptations. This approach reinforces that effective telerehabilitation requires redesigning clinical systems and care pathways, rather than digitizing in-person therapy content.

From a public health perspective, ATTEND-2 offers important implications for strengthening stroke rehabilitation in LMICs. Many LMICs face severe shortages of trained rehabilitation professionals and limited access to post-discharge therapy, particularly in rural areas. Platforms such as ATTEND-2 could complement these efforts by providing structured post-stroke rehabilitation pathways that extend care beyond hospital discharge. Integration of telerehabilitation within district hospital networks or community health programs may enable more equitable access to rehabilitation services for rural populations. Furthermore, ATTEND-2 aligns with India’s expanding digital health ecosystem, including the Ayushman Bharat Digital Mission. Policymakers could leverage such platforms to standardize post-discharge rehabilitation packages, particularly in underserved rural districts where workforce constraints are most pronounced.

A comparable model has been demonstrated through digital health platforms such as the Indian Control Hypertension Initiative app, which was developed to support screening of patients by CHWs using algorithm-based decision support [20]. In this model, CHWs conduct initial screening and provide basic management, while patients with uncontrolled risk factors or complications are referred to higher-level facilities or physicians. Similarly, the ATTEND-2 model could empower CHWs to strengthen post-stroke rehabilitation in resource-limited settings. Following an initial assessment, CHWs could facilitate and supervise basic rehabilitation exercises within the community, while physiotherapists remotely monitor progress and provide guidance through telerehabilitation. Such task-sharing models could improve access to rehabilitation services, particularly in rural areas, and align well with national digital health initiatives such as the Ayushman Bharat Digital Mission, by enabling continuity of stroke care beyond hospitalization. This approach is also consistent with the priorities outlined in the Rehabilitation 2030: A Call for Action initiative of the World Health Organization [21,22].

The intervention also offers a replicable framework for other LMICs facing similar rehabilitation-related barriers. Replication would require contextual adaptation such as local language translation, culturally relevant task selection, alignment with national telehealth regulations, and integration into country-specific financing mechanisms. The use of cross-platform development and Firestore cloud-based data storage enables real-time synchronization, secure data management, and scalability without requiring complex local IT infrastructure. Combined with minimal-equipment exercises, this design enhances portability across hospitals and community settings with differing levels of technological capacity. Additionally, the structured reporting using TIDieR-telehealth and CFIR domains enables systematic adaptation rather than ad hoc replication.

Strengths

The intervention may provide a scalable model for other LMICs facing similar workforce and infrastructure limitations. The use of smartphone-based platforms, minimal equipment requirements, and culturally adaptable exercise protocols makes the intervention transferable across diverse health systems. ATTEND-2 represents a pragmatic, implementation-oriented model that bridges evidence and delivery. By embedding behavioral science, digital infrastructure, and policy alignment within a structured reporting framework, it provides a transferable solution for expanding equitable stroke rehabilitation across India and other LMICs.

Limitations and future research directions section

The current report describes the design of the ATTEND-2 intervention. The feasibility and impact of the intervention on functional recovery, adherence, and long-term participation will require empirical evaluation through pilot and larger pragmatic trials (Appendix D presents the proposed CONSORT flow diagram for the planned ATTEND-2). In addition to the above-mentioned, further studies should include mobile internet connectivity and data requirements associated with the platform, as these factors may influence feasibility and sustained engagement in resource-constrained settings. 

Implementation risks remain, including inequities in smartphone access, variability in internet connectivity, shared device privacy concerns, and clinician workload redistribution. These factors may influence scalability and sustained implementation, particularly in rural and resource-constrained settings, and will require further evaluation during future feasibility and implementation phases. Long-term sustainability will depend on endorsement by health leaders and policymakers, financing integration, and measurable improvements in functional outcome.

## Conclusions

This paper provides a description of the ATTEND-2 app-based telerehabilitation for post-stroke upper limb rehabilitation in India, reported using the TIDieR checklist. By detailing the intervention components, this report aims to enhance clarity, replicability, and adaptation in similar contexts. ATTEND-2 illustrates a contextually tailored telerehabilitation model that integrates remote supervision, motivation, and safety within existing healthcare systems.

## Figures and Tables

**Figure 2:**
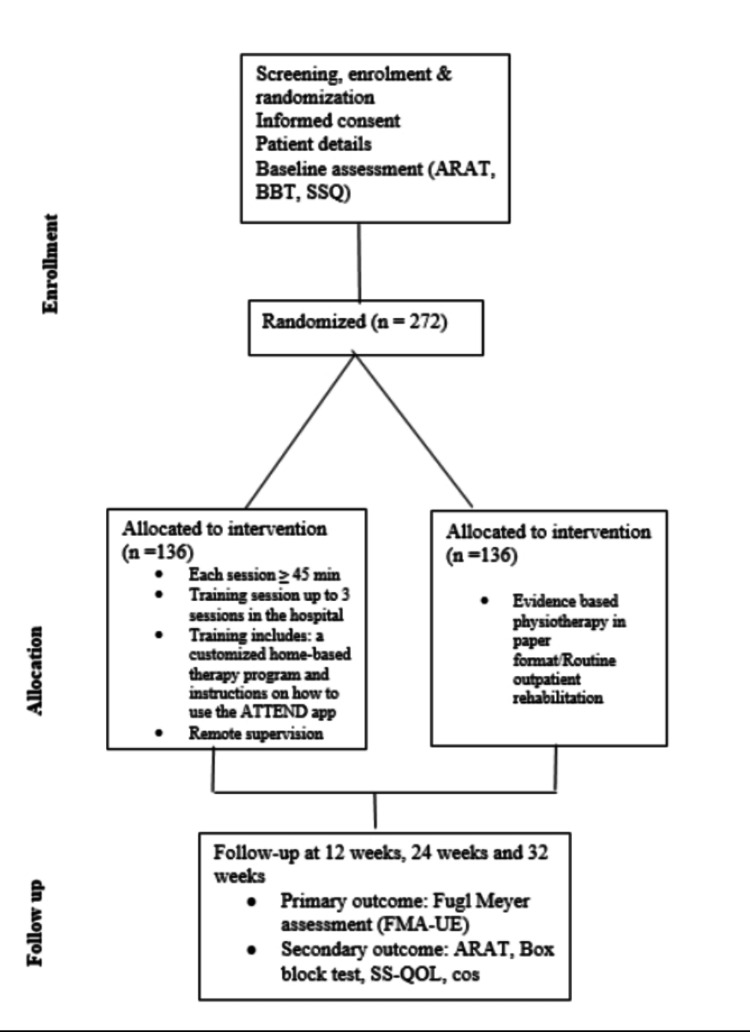
CONSORT Flow Diagram of Planned Participant Recruitment and Follow-Up

## Appendices

Appendix A 

**Table 2:** CFIR (Updated) Framework for ATTEND-2 Telerehabilitation Intervention in India

Innovation Domain: Smartphone-based telerehabilitation application for upper limb stroke recovery	
Construct name	ATTEND-2 app
A. Innovation Source	Developed collaboratively by stroke survivors, caregivers, physiotherapists, neurologists, and software developers.
B. Evidence Base	Informed by published literature, the ATTEND manual, prior Indian stroke rehabilitation exercise sets, doctoral research physical activity lists, and home-based rehabilitation protocols.
C. Innovation Relative Advantage	Provides low-cost, home-based rehab with remote supervision; addresses gaps in clinic access and the shortage of trained professionals in India.
D. Innovation Adaptability	Exercises categorized by FMA-UE severity and modified for small/shared Indian household spaces; instructions in English and regional languages (Punjabi, Kannada).
E. Innovation Trialability	Tested through an acceptability survey. Second acceptability survey and a 6-week feasibility pilot underway
F. Innovation Complexity	Moderate; requires managing digital platform, asynchronous exercise logs, and synchronous video consultations across Patient/Therapist/Admin modules.
G. Innovation Design	Progress dashboard with plant-growth metaphor, SOS icons, familiar household objects (bottles, towels), and culturally contextualized video demonstrations by stroke patients, remote monitoring by a therapist.
H. Innovation Cost	Free during trial; minimal operational costs for cloud hosting and maintenance.
Outer Setting: Home-based and community rehabilitation services	
A. Critical Incidents	None reported; system is resilient to minor technical disruptions.
B. Local Attitudes	Exercises are feasible and culturally appropriate; designed to encourage patient engagement.
C. Local Conditions	Addresses urban-rural differences, limited household space, and variable internet connectivity.
D. Partnerships & Connections	Collaborates with hospitals, rehabilitation centers, and professional networks for referrals and support.
E. Policies & Laws	Aligns with Indian national stroke care priorities, digital health policies, and hospital accreditation standards.
F. Financing	App development supported through research grants- WSO grant
G. External Pressure 1. Societal Pressure 2. Market Pressure 3. Performance Measurement Pressure	Adoption driven by limitations of generic stroke rehab apps that poorly addressed Indian patients’ needs. Performance pressures from national stroke guidelines, hospital accreditation standards, and the need for contextually relevant rehab programs motivated uptake. Limited mass media advocacy, but growing awareness of telerehabilitation as an essential post-stroke support. Minimal competition; existing apps not culturally tailored for Indian populations. Adherence to stroke care benchmarks and quality indicators influenced implementation.
Inner Setting: Christian Medical College, Ludhiana (CMCL)/ host institute	
A. Structural Characteristics	Requires secure cloud backend (Firebase), high-speed internet, and telehealth-equipped clinician workstations.
1. Work Infrastructure	Integration of telehealth tasks into therapists’ and doctors’ workflows; remote monitoring of patients and logs.
B. Relational Connections	Multidisciplinary teams (therapists, doctors, nurses) with strong professional networks.
C. Culture	Recipient-centeredness, deliverer-centeredness, and learning-centeredness; values data-driven practice and patient equity.
D. Compatibility	Aligns with continuum of stroke care in hospital and community settings.
E. Tension for Change	Hospital/OPD-based rehab limited; a shortage of trained professionals necessitated telerehabilitation with remote monitoring.
F. Relative Priority	Stroke telerehabilitation is considered a high-priority innovation alongside other digital health initiatives.
G. Incentive Systems	Patient outcomes, and research recognition motivate staff engagement.
H. Mission Alignment	Commitment to evidence-based, patient-centered stroke care.
I. Available Resources	Smartphones for patients/caregivers; clinician workstations; exercise materials.
J. Access to Knowledge & Information	Structured training provided for therapists and administrative staff (app navigation, monitoring).
Individuals Domain Roles & Characteristics (COM-B model):	
A. High-level Leaders	Hospital directors, department heads (Neurology/PMR).
B. Mid-level Leaders	Unit supervisors, senior therapists coordinate patient care.
C. Opinion Leaders	Experienced clinicians influencing adoption within teams.
D. Implementation Facilitators	Research staff and senior therapists supporting training and troubleshooting.
E. Innovation Deliverers	Physiotherapists, occupational therapists, and nursing staff.
F. Innovation Recipients	Adult stroke survivors and caregivers (co-deliverers).
G. Implementation Leads	Research team managing intervention delivery and adherence.
H. Implementation Team Members	Collaborative teams supporting therapists and patients.
I. Other Implementation Support	Administrative staff handling data and monitoring adherence.
Characteristics: Need: Stroke survivors have Upper limb functional deficits addressed by exercises. Capability: Therapists trained in ATTEND 2 and remote assessment. Opportunity: Access to smartphones, the internet, and caregiver support. Motivation: Driven to improve patient outcomes and adherence to standards.	
Implementation Process Domain: Structured, mixed-methods feasibility pilot.	
A. Teaming	Multidisciplinary coordination among therapists, doctors, and caregivers.
B. Assessing Needs	2 rounds of an acceptability survey with stroke stakeholders. 1 survey
C. Assessing Context	Urban-rural differences, household constraints, digital literacy, and available resources.
D. Planning	Roles, steps, milestones, and outcome measures defined using TIDieR and NROL frameworks.
E. Tailoring Strategies	Exercise selection, intensity, and frequency adjusted based on FMA-UE severity and patient feedback.
F. Engaging	Patient recruitment via hospital referrals; caregivers engaged to support home-based adherence.
G. Doing	Six-week intervention combining asynchronous exercises and brief synchronous check-in calls.
H. Reflecting & evaluating	Regular review of adherence, performance dashboards, and over video calls
I. Adapting	Modifications to exercises and app features planned post 2^nd^ acceptability and -6-week pilot based on outcomes and feedback.

Appendix B 

**Table 3:** Contextualized Exercises for Moderate (FMA 27–55) Post-Stroke Upper Limb Impairment

	Exercise	Description	Position	FITT	Material required
1	Large beads threading	Thread large beads onto a string	Sitting at the table	10–15 beads x 3 reps	Large beads, strings
2	Crumbling tissue paper	Crumple paper using one hand at a time	Sitting	10–15 reps x 3 reps	Tissue
3	Tearing paper strips	Tear paper using both hands or the affected hand only	Tabletop seated	Daily, moderate, 5–10 mins	Paper
4	Drawing shapes	Use crayons/pencils to draw circles, lines	Seated with support	10- 15 shapes x 3 sets	Crayon/ pencil, paper
5	Grasp a pen/pencil	Practice gripping and releasing a writing pen/pencil	Seated	10- 15 reps x 3 sets	Pencil/ pen
6	Opening and Closing Boxes	Use your affected hand to grasp, open, and close the lids repeatedly. If needed, stabilize the box with your unaffected hand.	Sitting	10- 15 reps x 3 sets	Boxes
7	Quadripod reach & grasp	Place objects in front of you. While weight bearing on the affected hand, reach targets	Prone	10- 15 reps x 3 sets	Target's box/deodorant/ tumbler
8	Folding a Bed sheet	Fold and tuck a bed sheet on a mattress or table, emphasizing the use of the affected hand to initiate the folding movement.	Sitting/Standing Using both hands, the person will smooth, fold, and pleat a bedsheet.	10-15 minutes 3 to 5 times per week	Bedsheet
9	Button/Unbutton a shirt	The person manipulates buttons through holes using both hands or one hand, with a focus on the affected hand while the sound hand provides support.	Seated comfortably at a table or standing	Daily or 3–5 times per week. 5–10 minutes per session or 5–10 repetitions per hand. Start with large buttons and progress to small, tight ones; vary speed and hand used	Buttons
10	Utensil washing using sponges, water, and soap	Focusing more on using the affected hand, the participant washes plates, cups, or spoons in a basin or sink. This involves reaching, scrubbing, rinsing, and stacking."	Standing at a sink or seated at a table with a basin	3–5 times per week or as part of ADL training.10–20 minutes per session	Utensils, sponge, dishwasher/soap
11 A	Reaching the overhead shelf	Instruct the patient to reach for a box placed on an overhead shelf to simulate a real-life functional task.	Standing	10–15 repetitions × 2–3 sets	Box
11 B	Reaching the overhead shelf	In standing, position the patient in front of a cupboard or shelf. Ask them to hold a lightweight glass in the affected hand and raise the arm to place the glass on a shelf at waist level.	standing	10–15 repetitions × 2–3 sets	cupboard
12	Wall Ball Toss – Below Shoulder Level	Patient tosses the ball to a spot on the wall at or below shoulder height and catches it	Standing/sitting	10 repetitions × 3 sets	Football
13	PNF with functional activity	Grasp an empty glass placed on the table using the affected hand and bring it toward the mouth. Progression: Fill the glass with water, and use different textures Or Instruct the patient to grasp a phone (or phone-like object) placed on the table using the affected hand and bring it toward the ear as if to take a call	Sitting	10 reps x 3 sets	Glass, table
14	ADL	Instruct the patient to hold a toothbrush with the affected hand and attempt to bring it to the mouth, simulating brushing movement.	Sitting/Standing	10 reps x 3 sets	Phone
15	ADL	Instruct the patient to grasp a comb with the affected hand and attempt to comb their hair	Standing	10 reps x 3 sets	Comb
16	Grocery bag	Ask the patient to lift a small shopping bag using a hook grasp (fingers only, no thumb). Start with an empty bag. Progress: add lightweight items like fruits or vegetables.	Standing/Sitting	10 reps x 3 sets	Bag
17	Door opening and closing	Instruct the patient to open and close a horizontal door handle or drawer handle using the hook of the fingers. Emphasize wrist neutral position and no thumb use.	Standing	10 reps x 3sets	Door with handle

Appendix C 

**Table 4:** Contextualized Exercises for Severe (FMA 0–27) Post-Stroke Upper Limb Impairment

	Exercise	Description	Position	FITT	Material required
1	Quadripod	Lie on your stomach, and slowly come on your knees and hands Progression: Increase the duration of hold time	Quadripod	Hold 30 seconds each	-
2	Mirror therapy	Place the affected arm behind the mirror and the unaffected limb in front of the mirror Move the unaffected limb while watching in the mirror. Include ADLs, opening and closing of a bottle and folding hand towel	Seated at a table with a mirror	Daily, 1–2 sets, 20 min sessions	Face Mirror, bottle, hand towel
3	Wand exercise	Use a wand/PVC pipe to guide the affected arm in movement (flexion/ abduction). If the patient is unable to grasp the wand/PVC pipe tie/hold the unaffected hand	Supine or seated	15 reps x 3sets	PVC pipe
4	Arm cradling	Use unaffected arm to support and move the affected arm in flexion, abduction	Sitting	15 reps x 3sets	-
5	Tabletop gliding	Glide your hand on a table with a towel, to reduce friction add talcum powder Progression: Don’t add talcum powder	Seated, elbow on the table	15 reps x 3sets	Hand towel
6	Rolling a wooden pin back and forth using palms or fingers	A person uses a wooden rolling pin to roll back and forth on a flat surface to flatten clay or dough, using the affected hand to move the rolling pin.	Seated at a table with forearms supported, or standing at a table.	3–5 sessions per week.5–15 minutes per session	Wooden pin
7	Seated Lat Push-Ups	While seated on a firm chair with armrests, instruct the patient to press down through their hands on the armrests, attempting to lift their body slightly or just engage the back and shoulder muscles (especially the lats). If lifting is too difficult, focus only on pushing effort and holding. Ensure trunk is upright.	Sitting on a chair with armrests, feet flat on the floor	10 reps × 3 set	Firm chair with armrests
8		While seated, hands clasped, bend forward with arms extended, and then raise your arms toward the ceiling without exceeding 90° if there's shoulder pain	Seated	10 reps × 3 set	-
9	Serratus Anterior Activation (Sitting)	In sitting, place a bottle in front of the patient. Instruct the patient to reach forward without bending the elbow, focusing on scapular protraction.	Sitting	10 reps x 3sets	Bottle
10	Eccentric Control of Shoulder External Rotators	In supine with shoulder abducted to 90°, place targets at intervals. Ask the patient to slowly lower their hand toward each bottle, boxes (into internal rotation) with control	Supine	10 reps x 3sets	Bottle/boxes
		With the shoulder positioned in 90° abduction and external rotation (like a "stop" sign), instruct the patient to bring their hand slowly to the mouth and return with control.	Supine	10 reps x 3sets	-
11	Elbow Flexion–Extension with Table Slide Support	The patient sits upright with the affected arm resting on a table, and a cloth or towel is placed under the hand to reduce friction. The task is to flex and extend the elbow, sliding the hand forward and backward along the table without using trunk movements (no leaning or compensatory shoulder hiking). Emphasis is on isolated elbow control in a supported position.	Sitting	10 reps x 3sets	Cloth/hand towel, table, chair
12	Wall targets	In a supine position, the patient supports the affected arm with the unaffected arm, maintaining it in shoulder flexion. Colored targets (e.g., sticky notes or visual markers) are placed on the wall within reachable range overhead by extending the elbow. The patient is instructed to reach toward each target one by one,	Supine	10 reps x 3sets	Colored targets/sticky notes
13	Straw Reach – Wrist Orientation Training	Tape a flexible straw to the distal part of the pronated forearm (back). Bend the straw tip away from the fingers. Instruct the patient to attempt to bring the wrist straw tip.	Sitting	10 reps× 3 sets	Straw

Appendix D 

Appendix E

**Table 5:** Contextualized Exercises for Mild (FMA 56–78) Post-Stroke Upper Limb Impairment

	Exercise	Description	Position	FITT	Material required
1	Towel squeezing	A towel is squeezed repeatedly	Seated or standing	10–15 squeezes x 3 reps	Towel
2	Lock and key	Hold the lock with the unaffected hand. With the affected hand, try to unlock	Seated at the table	10–15 reps x 3 reps	Lock and key
3	Shoe lacing	Hold the shoe with the unaffected hand if required. Pass the lace through all holes with affected handand tying shoelaces	Seated at the table	10–15 reps x 3 reps	Shoe with lace
4	Buttoning	Button and unbutton shirt/small cloth with the affected hand	Sitting at table	10–15 reps x 3 reps	Shirt with a button
5	Cloth pegs	Pinch and place clothespins along the edge of the cardboard with affected hand	Tabletop seated	10–15 reps x 3 reps	Cloth pegs
6	Coin stacking	Stack coins one on top of the other with affected hand	Seated at table	10–15 reps x 3 reps	Coin
7	Sorting grains	Separate grains like rice and lentils using a with affected hand	Tabletop seated	10–15 reps x 3 reps	Lentils, dal, rice etc
8	Pour water	Pour water from an empty bottle to glass with the affected hand Progress: half filled water to full	Seated or standing	10–15 reps x 3 reps	Bottle and glass
9	Mopping the floor using a traditional mop or microfiber stick	Using both hands, the participant engages in systematic mopping movements over a specified area with more emphasis on the affected hand and use the sound hand for support.	Standing (with or without support), may include walking if the area is large	3–5 times per week.10–20 minutes per session, depending on the area cleaned	Mop
10	Pleating a saree or dupatta	The individual folds fabric (such as a saree or dupatta) into neat pleats using coordinated finger and hand movements of the affected hand.	Seated or standing, with the fabric on a table, lap, or held upright	3–5 sessions/week. 10–15 minutes or 3–5 pleating repetitions per session	Dupatta/ Saree
11	Tying a turban	The participant demonstrates or simulates the technique of pleating and wrapping a turban, utilizing the affected hand to lead while the non-affected hand follows.	Standing or seated in front of a mirror	3–5 sessions/week. 10–20 minutes per session or until the turban is tied successfully.	Turban cloth
12	Wall Ball Toss – Overhead	The patient tosses a small ball above head level against a wall and catches it with the same hand.	Standing/sitting	10reps x 3sets	Tennis ball
13		Place a rubber band around your fingers and thumb. Spread your fingers apart and then relax.	Sitting	10reps x 3sets	Rubber band
14		Hold the toothbrush with the non-affected hand. Squeezing toothpaste onto the brush	Standing	10reps x 3sets	Toothbrush and paste
15	Sorting cards	Instruct the patient to pick up playing cards one by one using a pad-to-pad grasp (thumb and fingertip) and sort them by color, suit, or number.	Sitting	10reps x 3sets	Playing cards/ Visiting cards
16	Pleating hair	Pleating hair by asking the patient to reach overhead and manipulate a soft ribbon or their hair. For those with shorter hair, use a ribbon clipped to a headband or cap	Sitting/standing	10reps x 3sets	Ribbon
17	Wall push-ups	The patient stands facing a wall at arm’s length. Place both hands flat on the wall at shoulder height. Instruct the patient to bend the elbows slowly and bring the body closer to the wall (like a mini push-up), then push back to the starting position.	Standing	10 reps x 3 sets	-

## References

[1] Feigin V, Stark B, Johnson C (2021). Global, regional, and national burden of stroke and its risk factors, 1990-2019: a systematic analysis for the Global Burden of Disease Study 2019. Lancet Neurol.

[2] Pandian JD, Sudhan P (2013). Stroke epidemiology and stroke care services in India. J Stroke.

[3] Gandhi DBC, Mascarenhas R, Zarreen S, Chawla NS, Pandian JD, English C, Solomon JM (2025). Bridging the gap: unique strategies to improve access and implementation of stroke rehabilitation in LMICs - a scoping review. Disabil Rehabil.

[4] Kalkonde YV, Deshmukh MD, Sahane V, Puthran J, Kakarmath S, Agavane V, Bang A (2015). Stroke is the leading cause of death in rural Gadchiroli, India: a prospective community-based study. Stroke.

[5] The ATTEND Collaborative Group (2017). Family-led rehabilitation after stroke in India (ATTEND): a randomised controlled trial. Lancet.

[6] Laver KE, Adey-Wakeling Z, Crotty M, Lannin NA, George S, Sherrington C (2020). Telerehabilitation services for stroke. Cochrane Database Syst Rev.

[7] Aljedaani B, Babar MA (2021). Challenges with developing secure mobile health applications: systematic review. JMIR Mhealth Uhealth.

[8] Sarfo FS, Ulasavets U, Opare-Sem OK, Ovbiagele B (2018). Tele-rehabilitation after stroke: an updated systematic review of the literature. J Stroke Cerebrovasc Dis.

[9] Sarfo F, Ulasavets U, Opare-Sem O, Ovbiagele B (2026). Tele-rehabilitation after stroke: an updated systematic review of the literature. J Stroke Cerebrovasc Dis.

[10] Sureshkumar K, Murthy GV, Munuswamy S, Goenka S, Kuper H (2015). 'Care for Stroke', a web-based, smartphone-enabled educational intervention for management of physical disabilities following stroke: feasibility in the Indian context. BMJ Innov.

[11] John D, Dutta Majumdar A, Pillai RN (2024). Health technology assessment for digital health technologies in India: a framework for action. Int J Technol Assess Health Care.

[12] Sriram V, Ramani S, Srinivas PN (2024). Health policy processes in India: institutions, interests, ideas and contemporary debates. Research Handbook on Health Care Policy.

[13] Rhon DI, Fritz JM, Kerns RD (2022). TIDieR-telehealth: precision in reporting of telehealth interventions used in clinical trials - unique considerations for the Template for the Intervention Description and Replication (TIDieR) checklist. BMC Med Res Methodol.

[14] Ackerley S, Wilson N, Boland P, Peel R, Connell L (2024). NeuroRehabilitation OnLine: description of a regional multidisciplinary group telerehabilitation innovation for stroke and neurological conditions using the Template for Intervention Description and Replication checklist. Digit Health.

[15] Reardon CM, Damschroder LJ, Ashcraft LE (2025). The Consolidated Framework for Implementation Research (CFIR) user guide: a five-step guide for conducting implementation research using the framework. Implement Sci.

[16] Yang CL, Waterson S, Eng JJ (2021). Implementation and evaluation of the virtual Graded Repetitive Arm Supplementary Program (GRASP) for individuals with stroke during the COVID-19 pandemic and beyond. Phys Ther.

[17] (2026). StrokeEd: PUSH-UL program. StrokeEd.

[18] Gnanaprakasam A, Karthikbabu S, Ravishankar N, Solomon JM (2023). Effect of task-based bilateral arm training on upper limb recovery after stroke: a systematic review and meta-analysis. J Stroke Cerebrovasc Dis.

[19] Joseph G, Bhatti N, Mittal R, Bhatti A (2025). Current application and future prospects of artificial intelligence in healthcare and medical education: a review of literature. Cureus.

[20] Thakre S, Anjankar A, Singh A, Kumar T (2022). National hypertension guidelines: a review of the India Hypertension Control Initiative (IHCI) and future prospects. Cureus.

[21] (2026). Digital India: Ayushman Bharat Digital Mission. https://www.digitalindia.gov.in/initiative/ayushman-bharat-digital-mission/.

[22] (2026). World Health Organization: Rehabilitation in health systems. https://www.who.int/publications/i/item/9789241549974.

